# Nature of cobalt species during the *in situ* sulfurization of Co(Ni)Mo/Al_2_O_3_ hydro­desulfurization catalysts

**DOI:** 10.1107/S1600577519002546

**Published:** 2019-04-26

**Authors:** Mustafa al Samarai, Christa H. M. van Oversteeg, Mario Ulises Delgado-Jaime, Tsu-Chien Weng, Dimosthenis Sokaras, Boyang Liu, Marte van der Linden, Ad M. J. van der Eerden, Eelco T. C. Vogt, Bert M. Weckhuysen, Frank M. F. de Groot

**Affiliations:** a Inorganic Chemistry and Catalysis, Debye Institute for Nanomaterials Science, Utrecht University, Universiteitsweg 99, 3584 CG Utrecht, The Netherlands; b Stanford Synchrotron Radiation Lightsource, SLAC National Laboratory, 2575 Sandhill Road, Menlo Park, CA 94025, USA; c ID26, European Synchrotron Radiation Facility (ESRF), 71 Avenue des Martyrs, 38000 Grenoble, France

**Keywords:** heterogeneous catalysis, hydro­desulfurization (HDS) catalyst systems, CoMoS structure, hydro­treating catalysts, X-ray absorption spectroscopy, resonant inelastic X-ray scattering (RIXS)

## Abstract

The evolution in local structure and electronic properties of cobalt was investigated during *in situ* sulfurization. Using a combination of 1*s* X-ray absorption (XAS) and 1*s*3*p* resonant inelastic X-ray scattering (RIXS), the valence, coordination and symmetry of cobalt ions were tracked in two cobalt-promoted molybdenum oxide precursors of the hydro­desulfurization catalyst system, namely Co–Mo/Al_2_O_3_ and Co–Ni–Mo/Al_2_O_3_. Extended X-ray absorption fine structure shows that the Co—O bonds were replaced with Co—S bonds as a function of reaction temperature. The cobalt *K* pre-edge intensity shows that the symmetry of cobalt was modified from Co^3+^ *O*_*h*_ and Co^2+^ *O*_*h*_ to a Co^2+^ ion where the inversion symmetry is broken, in agreement with a square-pyramidal site. The 1*s*3*p* RIXS data revealed the presence of an intermediate cobalt oxy-sulfide species. This species was not detected from XAS and was determined from the increased information obtained from the 1*s*3*p* RIXS data. The cobalt XAS and RIXS data show that nickel has a significant influence on the formation of the cobalt oxy-sulfide intermediate species prior to achieving the fully sulfided state at *T* > 400°C.

## Abstract 2

From 1*s* X-ray absorption and 1*s*3*p* resonant inelastic X-ray scattering, the valence, coordination and symmetry of cobalt ions were tracked in two cobalt-promoted molybdenum oxide precursors of the hydro­desulfurization catalyst system.

## 1. Introduction   

The development of novel hydro­desulfurization (HDS) catalysts has been one of the interesting research subjects in the field of heterogeneous catalysis because of the new environmental regulations to reduce sulfur emissions of transportation fuels (Eijsbouts *et al.*, 2008 ▸). Cobalt- and nickel-promoted molybdenum sulfide catalysts supported on porous oxides, such as alumina, have been used since the early years of the previous century. The active phase of this catalyst is assumed to consist of MoS_2_ slabs with cobalt and/or nickel decorating the edges. There are many factors that can affect the activity of a catalytic solid, *e.g.* the metal loadings, type of support and especially the synthesis conditions and treatment procedures employed (Ratnasamy *et al.*, 1980 ▸; Keely *et al.*, 1984 ▸; Topsøe, Clausen, Topsøe & Pedersen *et al.*, 1986 ▸; Topsøe & Clausen, 1986 ▸; Prins *et al.*, 1989 ▸; Breysse *et al.*, 1991 ▸; Chianelli *et al.*, 1994 ▸; Startsev, 1995 ▸). Numerous studies have attempted to explain the catalytic HDS process and describe the role of the involved promoters Co and Ni on the Al_2_O_3_-supported MoS_*x*_ phase. The Co–MoS_*x*_ structure originally proposed by Topsøe and co-workers and Grunwaldt *et al.* (2004) ▸ was also confirmed by Bouwens *et al.* (1990 ▸, 1991 ▸, 1994 ▸) and Crajé *et al.* (1991*a* ▸,*b* ▸). The catalytic performance of this active phase is determined by the structure of the oxide precursor species, which is strictly dependent on the preparation methods (Topsøe *et al.*, 1984 ▸; Kibsgaard *et al.*, 2010 ▸). Therefore, as a starting point it is crucial to understand the processes involved in the synthesis of the catalyst and especially the sulfurization/activation step. In the petrochemical industry, the oxide precursors are activated by the addition of sulfur-containing compounds, *e.g.* methyl sulfides, to the feedstock. However, usually a mixture of 10% H_2_S/H_2_ is used to sulfurize the (supported) oxide precursors. Among the many synthesis steps involved, the sulfurization process is very likely the step that determines the final structure and performance of the active Co–Ni–Mo/Al_2_O_3_ catalyst material. Following the analysis of Topsøe and Clausen, we start from the assumption that cobalt can occur in three forms in alumina-supported catalysts: (*a*) as cobalt ions on the edges of the MoS_2_ crystallites, (*b*) as Co_9_S_8_ particles and (*c*) as divalent cobalt ions at the surface or in the bulk of the alumina (Topsøe *et al.*, 1984 ▸). Several characterization studies focused on the changes in (cobalt-doped) molybdenum compounds during the sulfidation process (Scheffer *et al.*, 1984 ▸; Arnoldy *et al.*, 1985 ▸; Nicosia & Prins, 2005 ▸; Rochet *et al.*, 2016 ▸). Recently Šarić *et al.* performed detailed density functional theory calculations of the Co–MoS_*x*_ system (Šarić *et al.*, 2017 ▸, 2018 ▸). They calculated the structures of the edges, basal plane and corners of the MoS_2_ slabs and found that the HDS activity is related to the small energy differences between structures with or without a sulfur atom for the corners and the S edges, but not the Mo edges.

In this article, we study the valence, coordination and spin-state changes of cobalt in single (cobalt-) and double (cobalt-nickel-) promoted HDS catalysts, using a combination of 1*s* X-ray absorption (XAS) and 1*s*3*p* resonant inelastic X-ray scattering (RIXS). The 1*s* core levels of the 3*d* transition metal ions can be probed with hard X-rays (4–10 keV). In contrast to the main *K*-edge, which arises from the dipole allowed transition of 1*s* to 4*p*, the pre-edge involves mainly 1*s*3*d* quadrupole transitions. The *K* pre-edge energy position and intensity are sensitive to the metal oxidation state, the site symmetry, and the crystal-field splitting. However, because of their relatively low intensity and large core hole lifetime broadening, their analysis is often limited to the energy position and intensity of the pre-edge. In a 1*s*3*p* RIXS experiment, the resonant 1*s*3*p* X-ray emission is measured by tuning the energy of the incident energy to a 1*s* X-ray absorption edge. This effectively combines X-ray absorption and X-ray emission into a single experiment (Glatzel *et al.*, 2009 ▸; Kotani *et al.*, 2001 ▸). The RIXS process can be viewed as an inelastic scattering of the incident photon at a resonance energy of the metal ion and is theoretically described by the Kramers–Heisenberg formula (Sakurai, 1967 ▸; Rubensson, 2000 ▸). The spectral broadening of 1*s* XAS is determined by the lifetime of the 1*s* intermediate state (*L*_1*s*_), whereas the spectral broadening of the 1*s*3*p* resonant XES depends on the final state 3*p* lifetime (*L*_3*p*_) (Glatzel *et al.*, 2005 ▸; Hill *et al.*, 1998 ▸). Because *L*_1*s*_ is larger than *L*_3*p*_, this results in the observation of spectral features on the X-ray emission energy scale with sharper line width than in a conventional X-ray absorption spectrum.

Hard X-rays are able to penetrate solids and probe the bulk phase properties of the samples (van Bokhoven *et al.*, 2004 ▸; Rabe *et al.*, 2010 ▸; Szlachetko *et al.*, 2013 ▸). This allows the determination of the active sites within the bulk of the sample under *in situ* conditions. In this work, RIXS is used to study the composition of the Co–Mo/Al_2_O_3_ and Co–Ni–Mo/Al_2_O_3_ HDS catalysts as model systems to reflect the abilities of the RIXS technique to study other heterogeneous catalysts under *in situ* conditions. The purpose of the present work is to unravel the nature of the cobalt species in the precursor oxide of γ-Al_2_O_3_-supported Co–Mo and Co–Ni–Mo catalysts and to follow the changes in the local structure and electronic properties, during the *in situ* sulfurization with RIXS and XAS.

## 2. Materials and methods   

### 2.1. Sample preparation   

The Co–Mo/Al_2_O_3_ and Co–Ni–Mo/Al_2_O_3_ catalyst materials were both synthesized by incipient wetness impregnation of the γ-Al_2_O_3_ support (BASF Al-4184, surface area of 157 m^2^ g^−1^ and pore volume of 0.75 ml g^−1^) with a solution of metal salts. Aqueous solutions of ammonium heptamolybdate hexahydrate [AHM, (NH_4_)_6_Mo_7_O_24_·6H_2_O, Sigma Aldrich, ≥99.0%], cobalt nitrate hexahydrate [Co(NO_3_)_2_·6H_2_O, Acros Organics, 99+%] for Co–Mo/Al_2_O_3_ and additionally nickel nitrate hexahydrate [Ni(NO_3_)_2_·6H_2_O, Sigma-Aldrich, 99.999%] for Co–Ni–Mo/Al_2_O_3_ were co-impregnated to obtain weight loadings of 14 wt% for molybdenum and 5 wt% for both cobalt and nickel. Subsequently, both samples were dried at 60°C for 16 h then at 120°C for 1 h. To convert the impregnated metal precursors to their respective metal oxides, the samples were calcined at 450°C for 16 h. All drying and calcination steps were performed under a (75% N_2_)/(25% O_2_) flow (1 ml min^−1^). We note that the atomic Co/Mo ratio of 0.58 is higher than used in the industrial HDS catalysts. We used the increased amount of cobalt and nickel to increase the signal-to-noise of the X-ray spectroscopy experiments.

**Figure 1:**
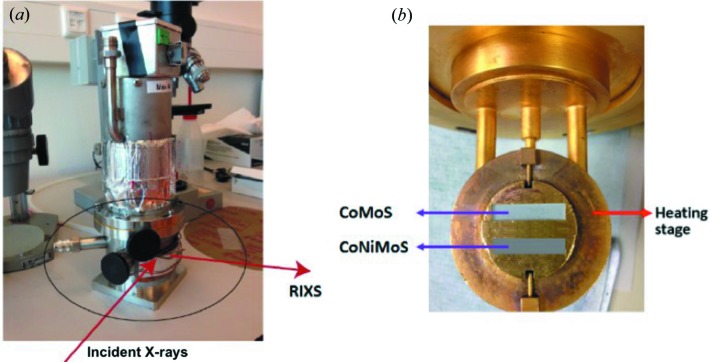
(*a*) Schematic of the dedicated sealed RIXS reactor showing the position of the incident X-rays and the scattered photons. The incident window was sealed with Kapton foil. The reactor has gas inlet (connected to a mass-flow controller) and outlet (to an exhaust stream) connections. Furthermore, to regulate the temperature the upper part of the reactor is connected to the temperature controller. (*b*) Overview of the sample holder with the sample pellets in the middle. The holder is placed in the heating stage.

During the *in situ* sulfurization experiments both oxide precursor samples were simultaneously sulfurized by loading the samples on the same sample holder. Pellets of Co–Mo/Al_2_O_3_ and Co–Ni–Mo/Al_2_O_3_ samples were made from the respective powders and loaded on the same sample holder as is shown in Fig. 1 ▸. Subsequently, the sample holder was placed inside the heating stage of the reactor. The sample was heated to 450°C with a ramp of 10°C min^−1^, while flowing with a 10% H_2_S/H_2_ mixture (1 ml min^−1^). The samples were kept at 450°C for 5 h.

The reactor was assembled and sealed and the incident window was covered with Kapton foil to decrease the photon flux and prevent radiation damage and leakage of air into the reactor. The reactor includes connections for both gas inlet and gas outlet. The inlet line is connected with a mass-flow controller that regulates the type and amount of gas that passes through the reactor. On the outlet side, there are bubbler airlocks that can be used to trap the toxic gas mixture. First, the oxide precursors were measured under ambient conditions, followed by *in situ* sulfurization under dynamic flow of 10% H_2_S/H_2_ gas mixture and by slowly heating the samples to 450°C.

### 2.2. X-ray experiments   

The spectra were recorded at beamline 6-2 of the Stanford Synchrotron Radiation Lightsource (SSRL) (Sokaras *et al.* 2013 ▸). The beamline is equipped with two double-crystal monochromators, Si(111) and Si(311). A collimating and a focusing Rh-coated mirror are positioned before and after the monochromator, respectively. The incident energy was selected using the Si(311) crystal during the measurements at the Co *K*-edge. After the monochromator, the beam is delivered to the Rh-coated focusing mirror and focused both horizontally and vertically to ∼140 µm × 400 µm (V × H) at the sample position. XAS spectra were measured simultaneously in total fluorescence yield (TFY) mode using a photodiode. The sample, crystal analyzer and photon detector were arranged in a vertical Rowland geometry. The Co *K*β (1*s*3*p*) RIXS spectra were recorded at a scattering angle of 90° in the horizontal plane using seven analyzer crystals (Bergmann & Cramer, 1998 ▸). The total energy resolution (Δ*E*/*E*) of the beamline and the spectrometer is ∼0.2 eV. The intensity was normalized to the incident flux. The RIXS data are shown as a contour map in a plane of incident and transferred photon energies, where the vertical axis represents the energy difference between the incident and emitted energies (energy-loss). The variations in colour on the plot correspond to the different scattering intensities.

The RIXS 2D maps were recorded in the excitation range 7704–7715 eV with steps of 0.2 eV, and 55 to 82 eV for the RIXS energy loss, with steps of 0.25 eV. The TFY XAS was also recorded with a photodiode. Radiation-damage studies were performed at ambient conditions by measuring four XAS spectra to check for any shifts in the edge jump energy, and the pre-edge intensity and its energy shift. Additionally, RIXS spectra were measured to reconfirm the results obtained with XAS. For all spectra, there was no change in the measured spectra for a 3 h experiment, confirming the absence of radiation damage.

## 3. Results   

### 3.1. Catalytic activity testing   

**Figure 2:**
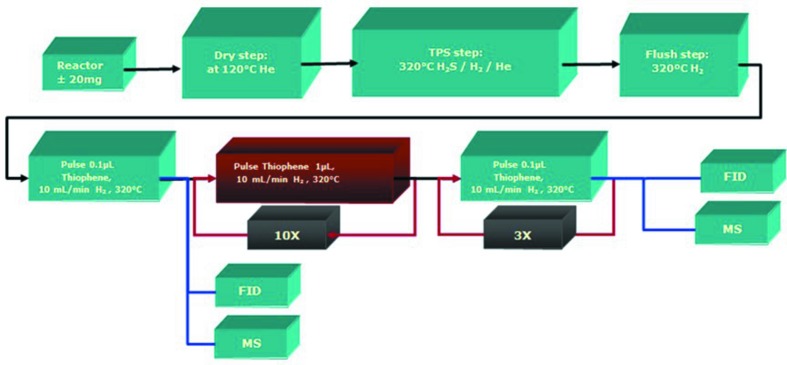
Schematic presentation showing the conditions used during catalytic thio­phene desulfurization activity tests of the freshly sulfided Co–Mo–S/Al_2_O_3_ and Co–Ni–Mo–S/Al_2_O_3_ catalyst material, during subsequent steps of the thio­phene HDS reaction.

**Figure 3:**
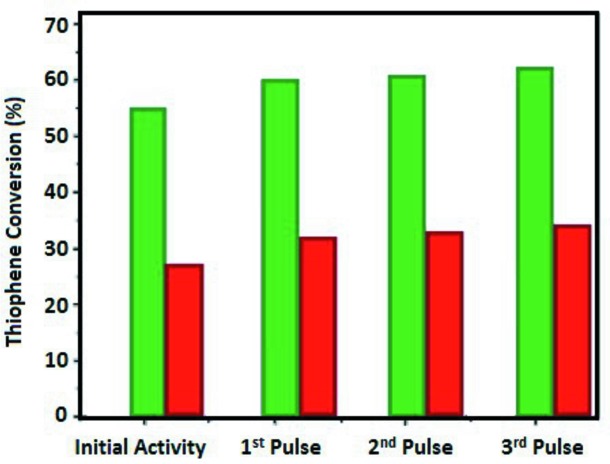
The normalized conversion of thio­phene by the Co–Mo–S/Al_2_O_3_ (green) and Co–Ni–Mo–S/Al_2_O_3_ (red) catalyst materials during three different desulfurization steps. For these catalytic tests (10%) thio­phene-saturated H_2_ flow was used.

Before presenting the results of the *in situ* sulfurization, we briefly discuss the catalytic activity of the Co–Mo–S/Al_2_O_3_ and Co–Ni–Mo–S/Al_2_O_3_ catalysts towards the thio­phene desulfurization. The HDS reaction of thio­phene was performed using thio­phene saturated H_2_ flow in a single-pass tubular down-flow fixed-bed reactor at the reaction conditions of 320°C. Fig. 2 ▸ shows a schematic summary of the performed catalytic studies. Prior to the reaction, the catalyst was reduced and sulfided for 6 h at 450°C at a heating rate of 5°C min^−1^ under a gas mixture containing 10 mol% H_2_S in H_2_ and a flow rate of 1 ml min^−1^. The initial activity test (the first pulse) was performed under 0.1 µl of thio­phene saturated in 10 ml min^−1^ H_2_, at a temperature of 320°C. The sample was then stabilized using 1 µl thio­phene saturated H_2_ with a flow rate of 10 ml min^−1^ at 320°C. Following a stabilization step, the catalytic activity of the samples was measured under similar conditions. The gaseous products were analyzed online on a mass spectrometer through a sampling and a flame ionization detector. Fig. 3 ▸ shows the normalized conversions of thio­phene by Co–Ni–Mo–S/Al_2_O_3_ and Co–Mo–S/Al_2_O_3_ during three subsequent HDS steps. Since variation in molybdenum-loading influences the HDS activity, the conversion is normalized for the molybdenum loading of the catalysts. An initial conversion of 27% was observed for the double promoted catalyst and 60% for the single promoted catalyst material. The thio­phene conversion data were normalized to the Mo loading.

Because of the higher ratio of promoters to molybdenum in Co–Ni–Mo–S/Al_2_O_3_, an increase in the formation of bulk cobalt- and nickel sulfide phases is expected, resulting in a reduced catalytic activity. The lower conversions observed for Co–Ni–Mo–S/Al_2_O_3_ can be explained by a decrease in the number of promoter atoms on the edges of MoS_2_ slabs. In addition, as shown in Fig. 3 ▸, an increase in the conversion rate was observed for the subsequent thio­phene pulses. This increase is caused by the correlation between the dispersion, degree of sulfidation of active phase and the HDS activity. Note that, because of the thio­phene treatment steps, the degree of sulfidation and dispersion of the active phase is increased.

### 3.2. XANES and EXAFS analysis   

**Figure 4:**
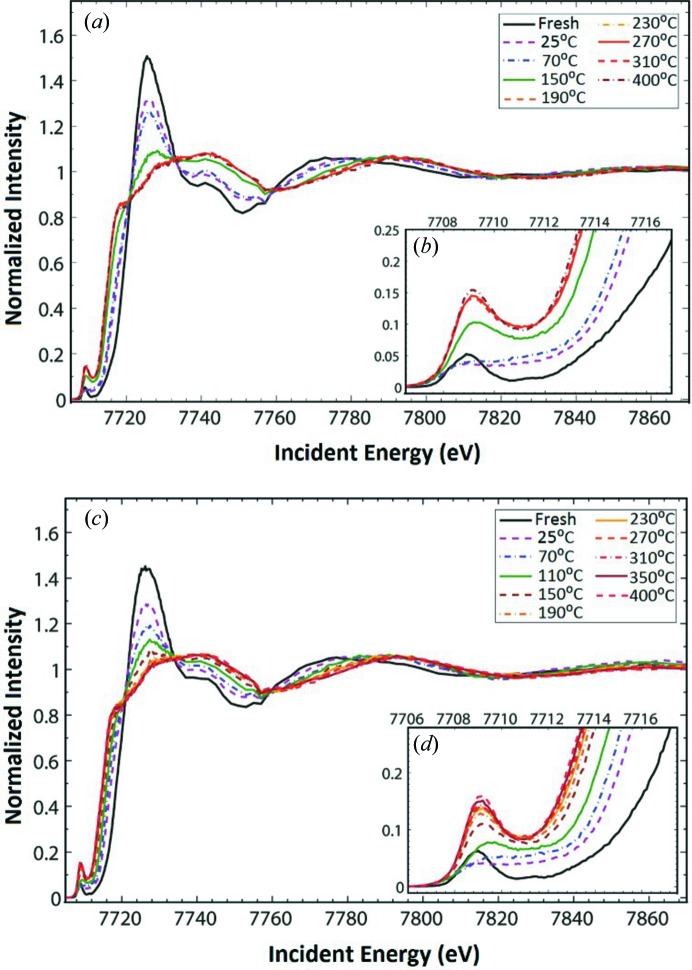
(*a*) Normalized Co *K*-edge XANES spectra for the Co–Mo/Al_2_O_3_ catalyst at various reaction temperatures and under an atmosphere of 10% H_2_S/H_2_; (*b*) inset of (*a*) around the pre-edge region. (*c*) Normalized Co *K*-edge XANES spectra for the Co-Ni-Mo/Al_2_O_3_ catalyst at various reaction temperatures and under an atmosphere of 10% H_2_S/H_2_; (*d*) inset of (*c*) around the pre-edge region.

The X-ray absorption spectra were normalized according to the procedure described in the supporting information. Fig. 4 ▸ shows the normalized cobalt *K*-edge X-ray absorption near-edge (XANES) region as a function of temperature. The cobalt *K*-edge spectrum of freshly calcined Co–Mo/Al_2_O_3_ is characterized by an intense main edge (1*s* → 4*p*) and a weak pre-edge feature with a main peak at 7709 eV followed by a weak shoulder at 7711.4 eV. This finding is similar to the previously reported Co_3_O_4_ reference (al Samarai *et al.*, 2016 ▸). Exposing both samples to the gas mixture at room temperature results in a significant shift of the main edge to lower energies implying a partial reduction of cobalt species accompanied by a decrease in the intensity of the pre-edge feature. In addition, the pre-edge intensity grows dramatically and the spectral shape is modified for both catalysts.

**Figure 5:**
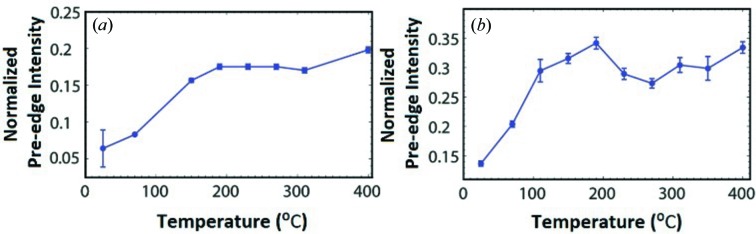
Pre-edge intensity areas of the Co *K*-edge XANES spectra of the (*a*) Co–Mo/Al_2_O_3_ and (*b*) Co–Ni–Mo/Al_2_O_3_ catalyst as a function of reaction temperature. If no error bars are given they are too small to be visible.

Upon heating the catalysts to higher reaction temperatures, the main edge shifts to lower energies, indicating a further reduction. In Fig. 5 ▸ the normalized intensity of the pre-edge of Co–Mo/Al_2_O_3_ and Co–Ni–Mo/Al_2_O_3_ shows an increase for *T* < 200°C, while for *T* > 200°C the pre-edge intensity is not further increased. The details of the fits including all numbers are given in Tables S2 and S3 of the supporting information. The increase in pre-edge intensity is assigned to a reduction in symmetry of Co^3+^ *O*_*h*_ and Co^2+^ *O*_*h*_ in the cobalt oxide species to a Co^2+^ ion in a square-pyramidal cobalt sulfide species for the fully sulfided catalyst sample (Borges *et al.*, 2012 ▸; Orita *et al.*, 2004 ▸; Paul *et al.*, 2008 ▸; Ma & Schobert, 2000 ▸). In this case, the crystal field splits the 3*d* orbitals partly belonging to the same irreducible representation of the point group as metal 4*p* orbitals, leading to partial mixing and hybridization (Griffith, 1964 ▸; Figgis, 1966 ▸). The increase in the pre-edge intensity is ascribed to the increase in the degree of the 3*d*4*p* mixing/hybridization character of the orbitals. We note that the pre-edge intensity is equivalent to that found in other studies (Nicosia & Prins, 2005 ▸; Rochet *et al.*, 2016 ▸) and lower than the pre-edge in Co_9_S_8_, which contains 50% tetrahedral and 50% octahedral sites. The observed pre-edge intensity indicates that if tetrahedral sites exist they relate to a maximum of 25% of the cobalt sites, the other 75% being octahedral.

In the performed composition analysis (listed in Tables S2 and S3), the Co–Mo/Al_2_O_3_ sample shows a higher rate of sulfidation compared with the Co–Ni–Mo/Al_2_O_3_ sample (Fig. 8). The Co–Mo/Al_2_O_3_ sample shows the complete sulfidation at 190°C, whereas the Co–Ni–Mo/Al_2_O_3_ sample is only fully sulfided at 400°C. Furthermore, these results are confirmed by the increase in the integrated pre-edge intensities and the analysis of the extended X-ray absorption fine structure (EXAFS) region during the sulfurization process, which was sensitive to mainly the presence of either Co—O or Co—S bonds. The EXAFS analysis illustrates the considerable impact that the temperature has on the sulfurization process and on the formation of Co—S bonds under the flow of the 10% H_2_S/H_2_ gas mixture (Fig. S6). At room temperature, approximately 45% and 50% of the total bonds are converted to Co—S bonds for Co–Mo/Al_2_O_3_ and Co–Ni–Mo/Al_2_O_3_ samples, respectively. This increase is attributed to gradual ligand substitution and conversion of cobalt ions at *O*_*h*_ sites in the case of the oxide precursor to the (*C*_4*v*_) square-pyramidal cobalt in symmetry for the fully sulfided sample.

### 3.3. 1*s*3*p* RIXS analysis   

**Figure 6:**
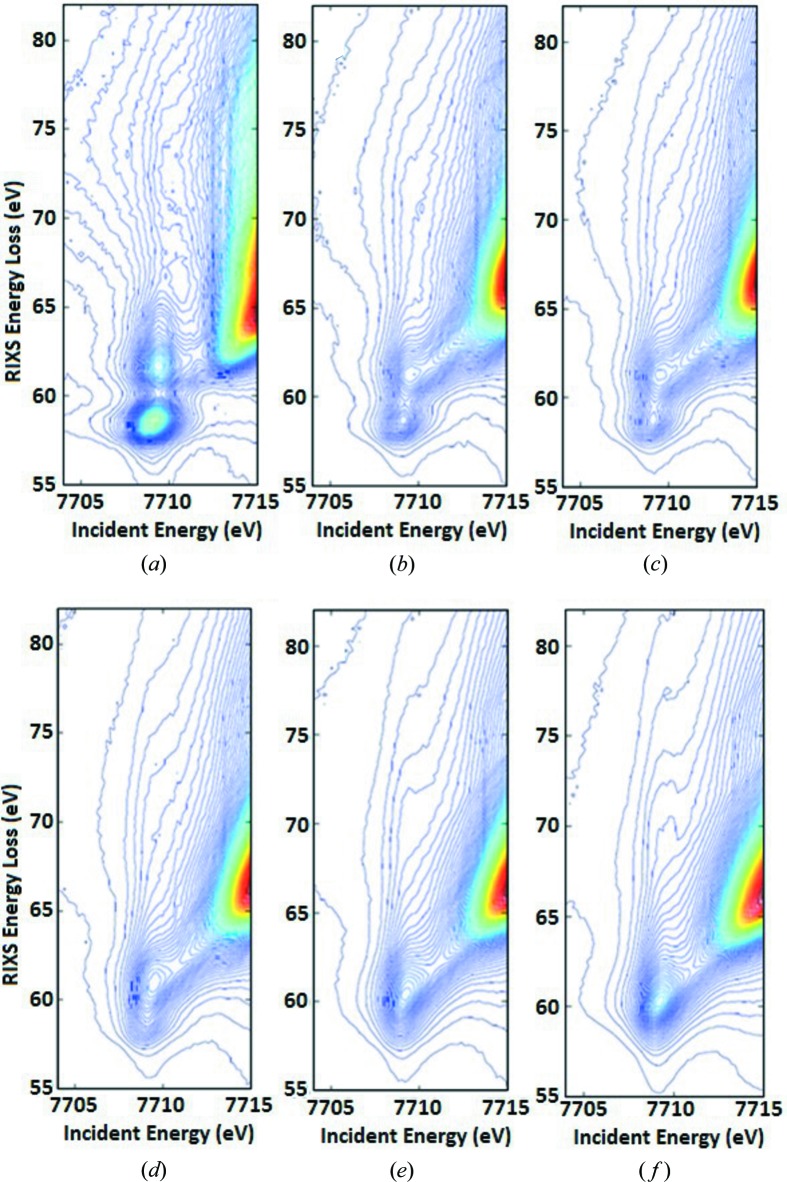
The experimental Co 1*s*3*p* RIXS data of Co–Mo/Al_2_O_3_. The freshly calcined sample was measured at 25°C (*a*). Subsequently we used gradual sulfidation by heating the sample under a dynamic 10% H_2_S/H_2_ gas mixture flow to 25°C (*b*), 70°C (*c*), 110°C (*d*), 150°C (*e*), and 400°C (*f*).

A series of cobalt 1*s*3*p* RIXS planes were recorded during the *in situ* sulfurization of Co–Mo/Al_2_O_3_ and Co–Ni–Mo/Al_2_O_3_. Fig. 6 ▸ shows the temperature-dependent 1*s*3*p* RIXS spectra of Co–Mo/Al_2_O_3_. The equivalent 1*s*3*p* RIXS spectra of Co–Ni–Mo/Al_2_O_3_ are shown in Section S3. The figure shows several changes during the *in situ* sulfurization reaction, including (*a*) the gradual conversion of two distinct pre-edge features to a single emission feature and (*b*) a shift of the main edge to lower absorption energies.

**Figure 7:**
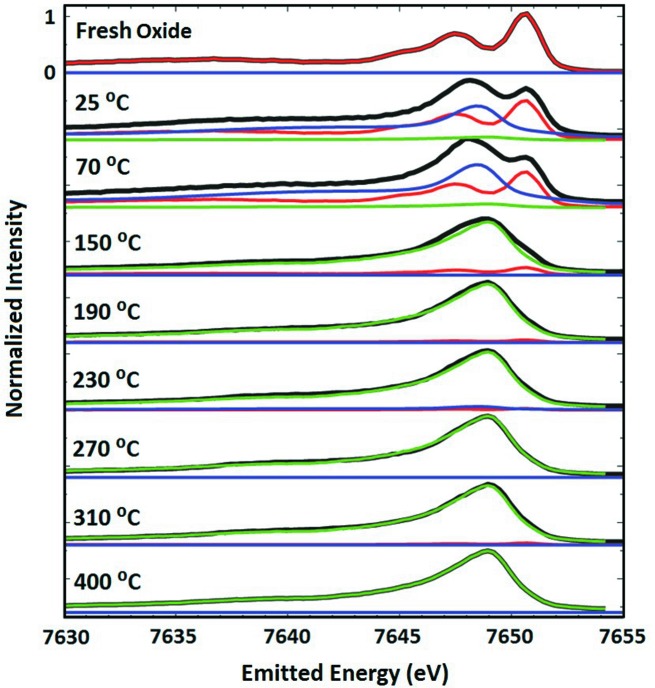
Resonant X-ray emission spectra (black) Co–Mo/Al_2_O_3_ obtained as a vertical cross-section through the pre-edge maximum (7709 eV) in Fig. 6 ▸. The character of the freshly calcined oxide sample is identified as 100% oxide (red) while the sulfided sample at 400°C shows a 100% sulfided character (green). The spectrum of the intermediate species is also included in the figure (blue).

For every RIXS plane the emission slice at the pre-edge peak position of 7709 eV was measured as shown in Fig. 7 ▸. The equivalent graphs of the Co–Ni–Mo/Al_2_O_3_ catalyst are shown in Figs. S4 and S5. The oxide precursor has distinctive resonance peaks at 7650.7 eV (peak 1) and 7647.45 eV (peak 2) characteristic of divalent cobalt species (al Samarai *et al.*, 2016 ▸). Subsequently, in the following steps, as the sample was exposed to the gas mixture, the RIXS spectra were affected and the resonant emission intensity ratios of the two peaks were reversed with peak 1 at 7648.15 eV and peak 2 at 7650.65 eV. This change can be ascribed to partial sulfidation of cobalt oxide species leading to modification in the symmetry of Co^2+^ ions.

The RIXS spectra were fit according to a procedure that involved a linear combination of the RIXS data on the freshly calcined sample (assumed to be in its oxide form) and the sample at 400°C for each temperature in addition to a small offset value to account for the counts on the tails of other emission processes. Initially, to fit the RIXS data, the combination of only two components was used to analyse the spectra. However, this proved to be impossible as additional peaks were observed in the experimental spectra during the *in situ* reaction. Therefore, additional empirical peaks were added to the model and collectively denoted as ‘intermediate species’. However, we imposed an important constraint to these empirical peaks: the broadening, shape energy position and relative intensities were forced to be the same in all spectral series (for all temperatures) by holistically fitting all spectra. We performed 100 fits in each stage of the *in situ* reaction using the methodology implemented in *Blueprint XAS* to explore uncertainty of all fit parameters (Delgado-Jaime *et al.*, 2010 ▸; Delgado-Jaime & Kennepohl, 2010 ▸). We then selected the best fits on the basis of the lowest sum squared error (SSE).

### 3.4. Combined analysis of XANES, EXAFS and RIXS   

**Figure 8:**
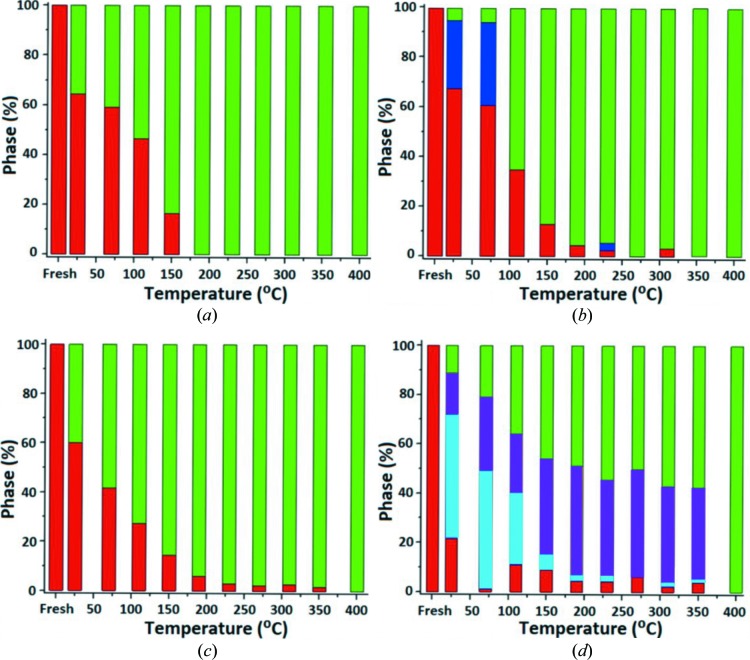
The phase conversion of cobalt species during the *in situ* sulfurization of Co–Mo/Al_2_O_3_ obtained by XANES (*a*) and RIXS (*b*). The Co–Ni–Mo/Al_2_O_3_ phase analysis by XANES (*c*) and RIXS (*d*) shows the presence of stable intermediates until the fully sulfided phase is obtained. In this figure, the oxide phase is indicated in red, while the fully sulfided phase is shown in green. The collection of intermediate cobalt oxysulfide species are indicated by the blue and purple.

The obtained phase concentrations are shown in Fig. 8 ▸. In the case of the Co–Mo/Al_2_O_3_ sample the RIXS data analysis revealed the presence of a single intermediate species (in blue) for the lower temperatures (*T* < 150°C). Because of its spectral shape, we can assume a species consisting mostly of Co—S bonds. However, the fitting of the Co–Ni–Mo/Al_2_O_3_ RIXS data was only possible by the inclusion of two intermediate species (blue and purple), which remain present during the *in situ* reaction for *T* < 400°C. The shape of one of the species has a predominantly oxide character [blue in (*a*)], while the second has a rather sulfide character [purple in (*b*)]. This assumption is based upon their comparison with the spectra of the pure oxide and the pure sulfide with regard to their individual shape as well as the position and intensity of the spectral features. In other words, in Co–Mo/Al_2_O_3_ the intermediate is most likely to be the Co—S_5_O and/or Co—S_4_O_2_ oxy-sulfide, which can be distinguished from Co—S_6_ in some intermediate stages. In Co–Ni–Mo/Al_2_O_3_ we see both the Co—S_5_O and/or Co—S_4_O_2_ oxy-sulfide (blue) and also the Co—S_1_O_5_ and Co—S_2_O_4_ oxy-sulfides (purple).

The Co–Ni–Mo/Al_2_O_3_ sample is more sensitive toward sulfidation at room temperature as 60% (versus 30% for Co-Mo/Al_2_O_3_) of the oxide species is already converted to either the sulfide or intermediate species under the dynamic flow of the gas mixture at 25°C. The details of the analysis and all numbers are given in Tables S4 and S5. We note that at 400°C oxidic cobalt is no longer detected, implying the absence of divalent cobalt ions in the alumina. In addition, a fraction of the sulfide phase could exist as Co_9_S_8_ particles.

## 4. Discussion   

During the gradual sulfurization of cobalt in the Co–Mo/Al_2_O_3_ and Co–Ni–Mo/Al_2_O_3_ samples, an increase in the pre-edge intensity was observed. The increase is caused by the conversion of cobalt species to a species without inversion symmetry, for example in square-pyramidal symmetry. Furthermore, because of the sulfurization process the cobalt was reduced and the Co *K* main edge was also shifted to lower absorption energies. The EXAFS data analysis showed the gradual ligand exchange during the process of sulfurization. The relative concentration percentages of Co—O and Co—S bonds were determined by fitting the EXAFS data as the linear combination of components at the two extremes. The analysis showed a higher tendency towards sulfurization of cobalt in the case of Co–Mo/Al_2_O_3_. In addition, the gradual change in the composition was obtained for the subsequent steps of the *in situ* reaction.

**Figure 9:**
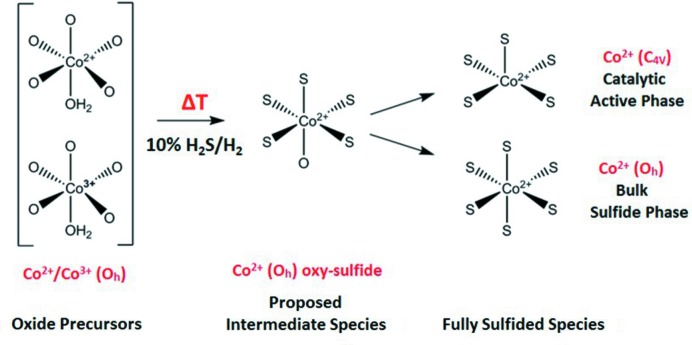
Representation of the proposed species involved during the *in situ* sulfurization of the freshly calcined Co–Mo/Al_2_O_3_ and Co–Ni–Mo/Al_2_O_3_. The oxide precursor was identified as octahedral Co^2+^ and Co^3+^. Prior to the complete sulfided state, cobalt oxy-sulfide intermediate species are formed. At 400°C there are two possible reaction pathways that result in either the catalytic active Co^2+^ (*C*_4*v*_) sulfide species or the bulk Co^2+^ (*O*_*h*_) sulfide species.

The Co 1*s*3*p* RIXS study has shown a gradual transition of the cobalt oxide in the active phase to the fully sulfided species. One of the findings is that the Co–Mo/Al_2_O_3_ catalyst has a higher tendency for the sulfurization reaction and achieves a >90% conversion to the sulfide phase at 190°C, compared with 400°C for the Co–Ni–Mo/Al_2_O_3_ catalyst. By analyzing the fits obtained from the RIXS data, the existence of a single intermediate species for *T* ≤ 150°C with a sulfide-like character was detected for the Co–Mo/Al_2_O_3_ catalyst. In the case of the Co–Ni–Mo/Al_2_O_3_ catalyst two intermediates were identified that eventually converted to the fully sulfided phase at 400°C. The RIXS data confirmed the previously obtained results by XANES and EXAFS analysis of the two samples. A summary of the proposed cobalt species involved during the *in situ* sulfurization of the Co–Mo/Al_2_O_3_ and Co–Ni–Mo/Al_2_O_3_ HDS catalysts is shown in Fig. 9 ▸. The two phases found in the fully sulfided phase are not in agreement with the recent studies of Šarić *et al.* (2017 ▸, 2018 ▸), who calculate the HDS activity as a difference between tetrahedral cobalt sites. As discussed in Section 3.2, the observed pre-edge intensity indicates that if tetrahedral sites exist they can relate to a maximum of 25% of the cobalt sites, the other 75% being octahedral. The results are in agreement with the alternative of the cobalt having square-pyramidal symmetry.

## 5. Conclusions   

We conclude that from a combination of cobalt 1*s* XANES, EXAFS and 1*s*3*p* RIXS we derived several boundary conditions regarding the mechanisms of sulfurization in Co–Ni–Mo/Al_2_O_3_ catalysts. EXAFS shows that the Co—O bonds are replaced with Co—S bonds as a function of reaction temperature, and the pre-edge intensity shows that the symmetry of cobalt is modified from Co^3+^ *O*_*h*_ and Co^2+^ *O*_*h*_ to a Co^2+^ ion where the inversion symmetry is broken, in agreement with a square-pyramidal site. The cobalt 1*s*3*p* RIXS reveals the presence of an intermediate cobalt oxy-sulfide species.

The catalytic data shows a large influence of nickel in the Co–Ni–Mo/Al_2_O_3_ catalyst in comparison with Co–Mo/Al_2_O_3._ The cobalt XAS and RIXS data show that nickel has a significant influence on the formation of the cobalt oxy-sulfide intermediate species prior to achieving the fully sulfided state.

## 6. Supporting information   

The supporting information is available as a PDF file containing information on (1) the catalytic activity testing, (2) the XAS data analysis method, (3) the numerical XANES fit results and (4) the 1*s*3*p* RIXS images of the CoNiMo catalyst.

## Supplementary Material

Supporting information . DOI: 10.1107/S1600577519002546/hf5380sup1.pdf
