# Supplementary material for: Nature of cobalt species during the in situ sulfurization of Co(Ni)Mo/Al2O3 hydro­desulfurization catalysts
Source: J Synchrotron Radiat. 2019 Apr 26;26(Pt 3):811–8. doi: 10.1107/S1600577519002546 (PMC6510205; doi:10.1107/S1600577519002546)
Supplement: Supplementary file 1 [file s-26-00811-sup1.pdf]

JOURNAL OF SYNCHROTRON RADIATION

Volume 26 (2019)

Supporting information for article:

Nature of cobalt species during the in-situ sulfurization of Co(Ni)Mo/Al2O3 hydrodesulfurization catalysts

Mustafa al Samarai, Christa H. M. van Oversteeg, Mario Ulises Delgado-Jaime, Tsu-chien Weng, Dimosthenis Sokaras, Boyang Liu, Marte van der Linden, Ad M. J. van der Eerden, Eelco T. C. Vogt, Bert M. Weckhuysen and Frank M. F. de Groot

S1. XAS Analysis: Fit Models and Methodology

This section describes the model used for the analysis of the x-ray absorption spectra, both x-ray absorption near edge (XANES) as the extended x-ray absorption fine structure (EXAFS) region.

The XAS data were normalized and analyzed for composition according to two sequential fitting procedures. In step one, a model to fit the EXAFS region of the freshly calcined and those of the samples at 400 °C (both CoMo/Al_2O_3 and CoNiMo/Al_2O_3) was used. The model contained the following features:

I. A pre-edge baseline that transitions into a spline after the edge;

II. A cumulative pseudo-Voigt function to account for the 1s-4p edge jump; and

III. Calculated paths modeling the back-scattering of the Co-S and Co-O bonds to fit the EXAFS of the fresh (freshly calcined oxide) and of the 400°C (fully sulfided) forms. These paths are calculated from FEFF. Parameters such as Bond distance, Debye-Waller, and E0 were allowed to float during the fitting.

We assumed an octahedral geometry for the oxide and a square-pyramidal for the sulfide, but as discussed below, the spectra above 7750 eV are relatively insensitive to a specific geometry. The dominant paths for energies higher than 7750 eV was recognized as either the Co-O and Co-S bonds. Thus, we note that this region of the spectrum is not sensitive to a specific geometry, but instead to the presence or absence of Co-O and Co-S bonds.

The fitting of this model was performed simultaneously with an evolving k^3-weighted k-space. This evolving k-space data is useful because its form is modified according to the changing spline and the value of E_0, as the fitting progresses evolves. The main purpose of this step is to define a good spline and pre-edge background and to evaluate the edge intensity (for normalization purposes). However, in this step, the fit of the pre-edge features was ignored.

We attempted 100 fits in each case using the methodology implemented in Blueprint XAS to reduce bias and to explore uncertainty of all fit parameters. We then selected the best fits on the basis of their sum of square errors (SSE) in both energy and k-space. “SSE” is a network performance function and measures the performance according to the sum of squared errors.

The average of the results are shown in Figures S1 (for CoMo/Al_2O_3) and S2 (for CoNiMo/Al_2O_3) and their respective fit parameters are summarized in Table S1. We note that while the trends in E_0 and bond-distance are consistent for CoMo/Al_2O_3 and CoNiMo/Al_2O_3 (depending on the type of bond), the absolute values are harder to establish from this analysis, as these two variables are strongly correlated (Figure S3). A good model that fits the near-edge region also can provide better estimates on the absolute E_0 values, and a better estimate on bond distances would require longer EXAFS regions. However, our main goal from this analysis is to collect a good collection of splines and corresponding edge intensities that we could use in the next step.

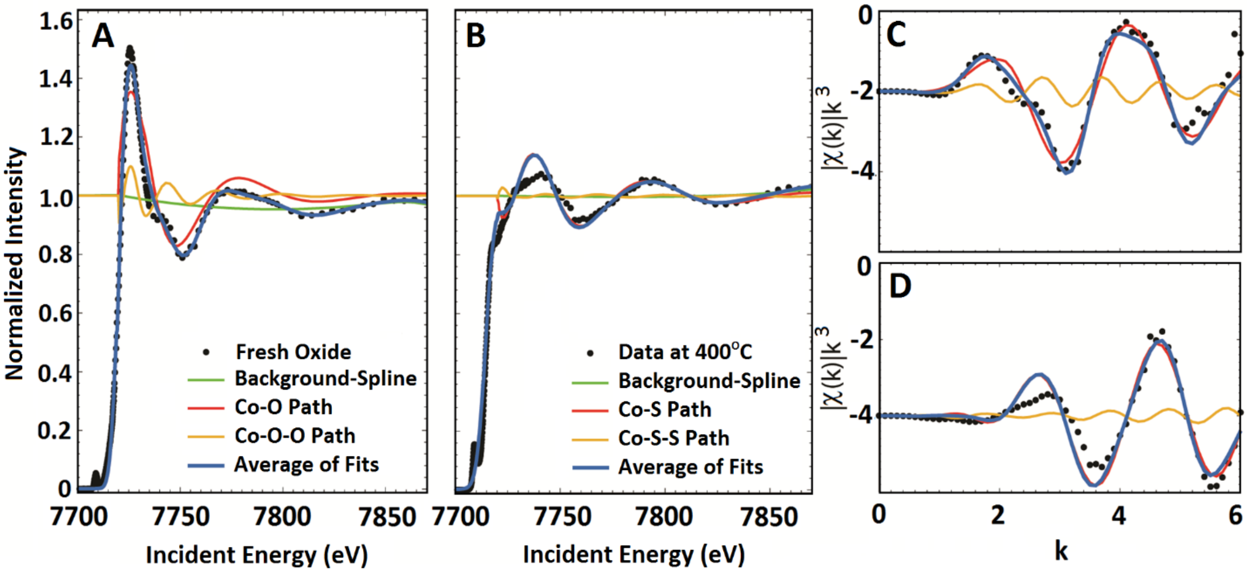

Figure S1 Average of fits to the data and corresponding components (normalized by the obtained edge intensity) for A) the freshly calcined sample of $CoMo/Al_2O_3$ and B) the data measured at 400°C. The corresponding average on the evolving k-space for A) and B) are shown in C) and D), respectively.

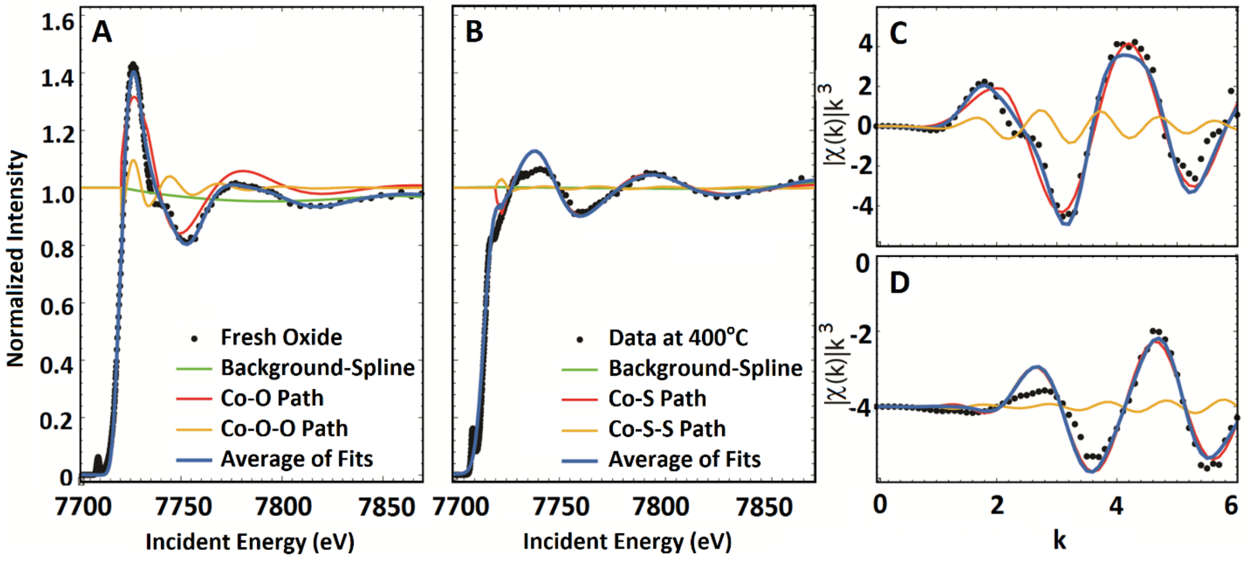

Figure S2 Average of fits to the data and corresponding components (normalized by the obtained edge intensity) for A) the freshly calcined oxide sample of $CoNiMo/Al_2O_3$ and b) the data measured at 400°C. The corresponding average on the evolving k-space for A) and B) are shown in C) and D), respectively.

Table S1 List of relevant parameters obtained from the fits in step one for the freshly calcined CoMo/Al_2O_3 and CoNiMo/Al_2O_3 and the corresponding data obtained at 400°C.

Data set	E_0	Bond distance Co-O/Co-S	Debye-Waller Co-O/Co-S	Debye-Waller Co-O-O/ Co-S-S
**CoMo/Al_2O_3-Freshly Calcined**	**7716.1 ± 0.1**	**1.98 ± 0.003**	**0.01 ± 0.001**	**0.003 ± 0.001**
**CoMo/Al_2O_3-400°C**	**7713.0 ± 3.4**	**2.18 ± 0.029**	**0.01 ± 0.001**	**0.002 ± 0.001**
**CoNiMo/Al_2O_3-Freshly Calcined**	**7716.6 ± 0.3**	**1.96 ± 0.050**	**0.01 ± 0.002**	**0.005 ± 0.001**
**CoNiMo/Al_2O_3-400°C**	**7713.4 ± 4.7**	**2.18 ± 0.038**	**0.01 ± 0.002**	**0.003 ± 0.008**

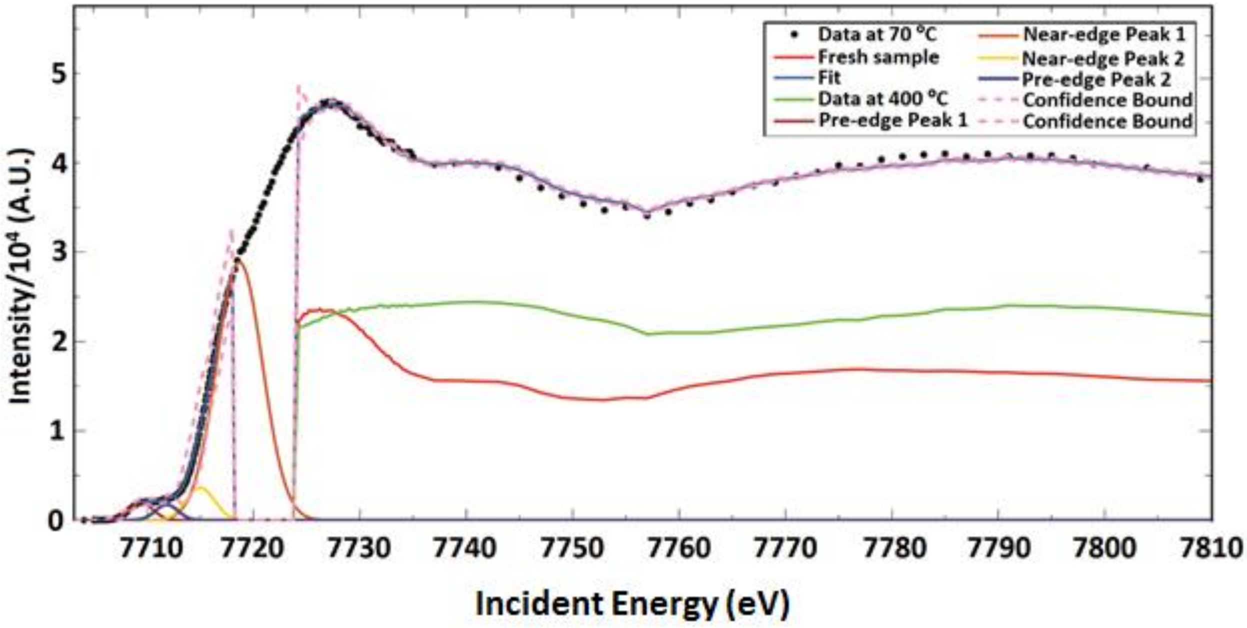

Figure S3 Representative fit showing the model implemented to analyze the XANES data of CoMo/Al_2O_3 and CoNiMo/Al_2O_3 as a function of temperature and under the exposure with 10% H_2S/H_2. For composition analysis, the experimental spectra of the freshly calcined (oxides) and those measured at 400 °C (assumed to be sulfides) are used as references above 7750 eV. The pre-edge is analyzed empirically by the inclusion of four peaks (two in the pre-edge and two more near the edge).

This model included the following features:

I. To evaluate the pre-edge areas, we included two pre-edge pseudo-Voigt peaks plus two additional peaks to model the near-edge region;

II. The composition was established as the linear combination of the XANES spectra of the freshly calcined sample and the sulfided sample at 400°C. This linear combination was set active only above 7725 eV with the use of a unit step function in order to allow the pre-edge area to be evaluated empirically. Because the start of the EXAFS region is mostly sensitive to one path, this linear combination can be also envisaged in terms of the amount of Co-S or Co-O bonds, independent to variations in the pre-edge, which should be more sensitive to electronic effects.

III. For normalization, the intensity of the two components (the freshly calcined oxide and the final sulfided sample at 400°C) are not known a priori and are dependent on the results of step one. Thus, an *arbitrary* intensity of $1x10^4$ (the same for both references) is initially assumed for these components.

IV. Each data set is then fit using this model based on the same methodology than in step one. Moreover, the good fits obtained for each data set were combined with the results obtained in the previous stage to create a grid where all the combinations of edge intensities and splines of both references are used to normalize the data and the pre-edge intensity, to recalculate compositions (based on *actual* intensities) and to reconstruct (based on the found compositions) backgrounds to subtract from the data sets.

Figures 3 and 4 show the average results from this procedure, whereas Figure 5 (and Tables S2 and S3) list the resulting normalized pre-edge area (first two pseudo-Voigt peaks). From Figures 3A and 4A we note the presence of isosbestic points, which confirm that this region involves only two species (the replacement of Co-O bonds by Co-S ones). This is not the case in the pre-edge (Figures 3B and 4B and Figure 5) and the changes on this region suggest a more complex evolution of species. A detailed analysis in this region is complicated because the features of all the possible species involved absorb in the same small energy region. Figure 8 shows the compositions expressed as the percentage of the spectrum on the freshly calcined sample and on the sample at 400°C obtained by XANES (Figure 8 A and C) and RIXS (Figure 8 B and D) for $CoMo/Al_2O_3$ and $CoNiMo/Al_2O_3$.

S2. The XANES fit results in table form.

Table S2 Obtained pre-edge intensities and compositions for $CoMo/Al_2O_3$ as a function of temperature and under and atmosphere of 10% H_2S/H_2, expressed in terms of the relative amounts of the spectrum of the freshly calcined sample and the one collected at 400°C and in terms of Co-O and Co-S bonds.

Data set	Pre-edge Area	% Fresh	% 400°C	% Co-O bonds	% Co-S bonds
Fresh Oxide	0.099 ± 0.001	100	0	100	0
25°C	0.128 ± 0.050	64.3 ± 0.9	35.7 ± 0.9	54.6 ± 0.1	45.4 ± 0.1
70°C	0.166 ± 0.001	59.1 ± 0.5	40.9 ± 0.5	49.1 ± 0.1	50.9 ± 0.1
150°C	0.313 ± 0.005	16.5 ± 0.3	83.5 ± 0.3	11.6 ± 0.1	88.3 ± 0.1
190°C	0.350 ± 0.007	<0.1	>99.9	<0.1	>99.9
230°C	0.350 ± 0.007	<0.1	>99.9	<0.1	>99.9
270°C	0.350 ± 0.007	<0.1	>99.9	<0.1	>99.9
310°C	0.340 ± 0.007	0.2 ± 0.1	99.8 ± 0.1	<0.1	>99.9
400°C	0.396 ± 0.008	0	100	0	100*

Table S3 Obtained pre-edge intensities and compositions for $CoNiMo/Al_2O_3$ as a function of temperature and under and atmosphere of 10% H_2S/H_2, expressed in terms of the relative amounts of the spectrum of the freshly calcined sample and the one collected at 400°C and in terms of Co-O and Co-S bonds.

Data set	Pre-edge Area	% Fresh	% 400°C	% Co-O bonds	% Co-S bonds
Freshly calcined	0.128 ± 0.005	100	0	100	0
25°C	0.186 ± 0.004	59.9 ± 1.0	40.1 ± 1.0	49.9 ± 0.1	50.1 ± 0.1
70°C	0.254 ± 0.005	41.6 ± 1.0	58.5 ± 1.0	32.2 ± 0.1	67.8 ± 0.1
110°C	0.344 ± 0.019	27.4 ± 0.8	72.6 ± 0.8	20.1 ± 0.1	79.9 ± 0.1
150°C	0.365 ± 0.008	14.5 ± 0.5	85.5 ± 0.5	10.2 ± 0.1	89.8 ± 0.1
190°C	0.392 ± 0.010	6.0 ± 0.2	94.0 ± 0.2	4.1 ± 0.1	95.9 ± 0.1
230°C	0.339 ± 0.009	3.0 ± 0.1	97.0 ± 0.1	2.1 ± 0.1	97.9 ± 0.1
270°C	0.323 ± 0.008	2.1 ± 0.1	97.9 ± 0.1	1.4 ± 0.1	98.6 ± 0.1
310°C	0.354 ± 0.013	2.6 ± 0.1	97.4 ± 0.1	1.8 ± 0.1	98.2 ± 0.1
350°C	0.348 ± 0.020	1.7 ± 0.1	98.3 ± 0.1	1.2 ± 0.1	98.8 ± 0.1
400°C	0.384 ± 0.010	0	100	0	100*

S3. 1s3p RIXS images of CoNiMo/Al_2O_3

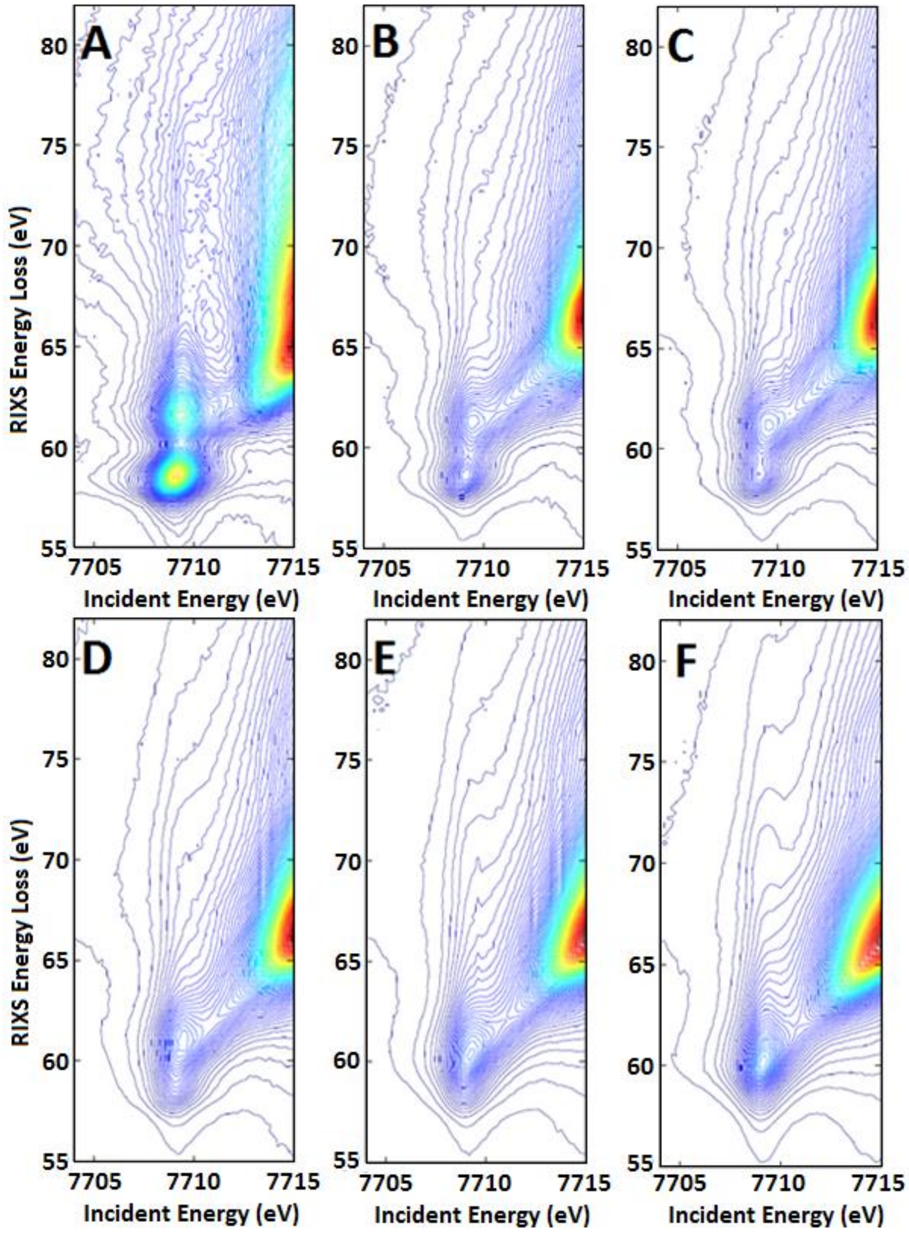

Figure S4 The experimental Co 1s3p RIXS data of CoNiMo/Al_2O_3. In a first step the freshly calcined precursor was measured (A) followed by gradual sulfidation by heating the sample under a dynamic 10% H_2S/H_2 gas mixture flow to 25°C (B), 70°C (C), 110°C (D), 150°C (E), and 400°C (F).

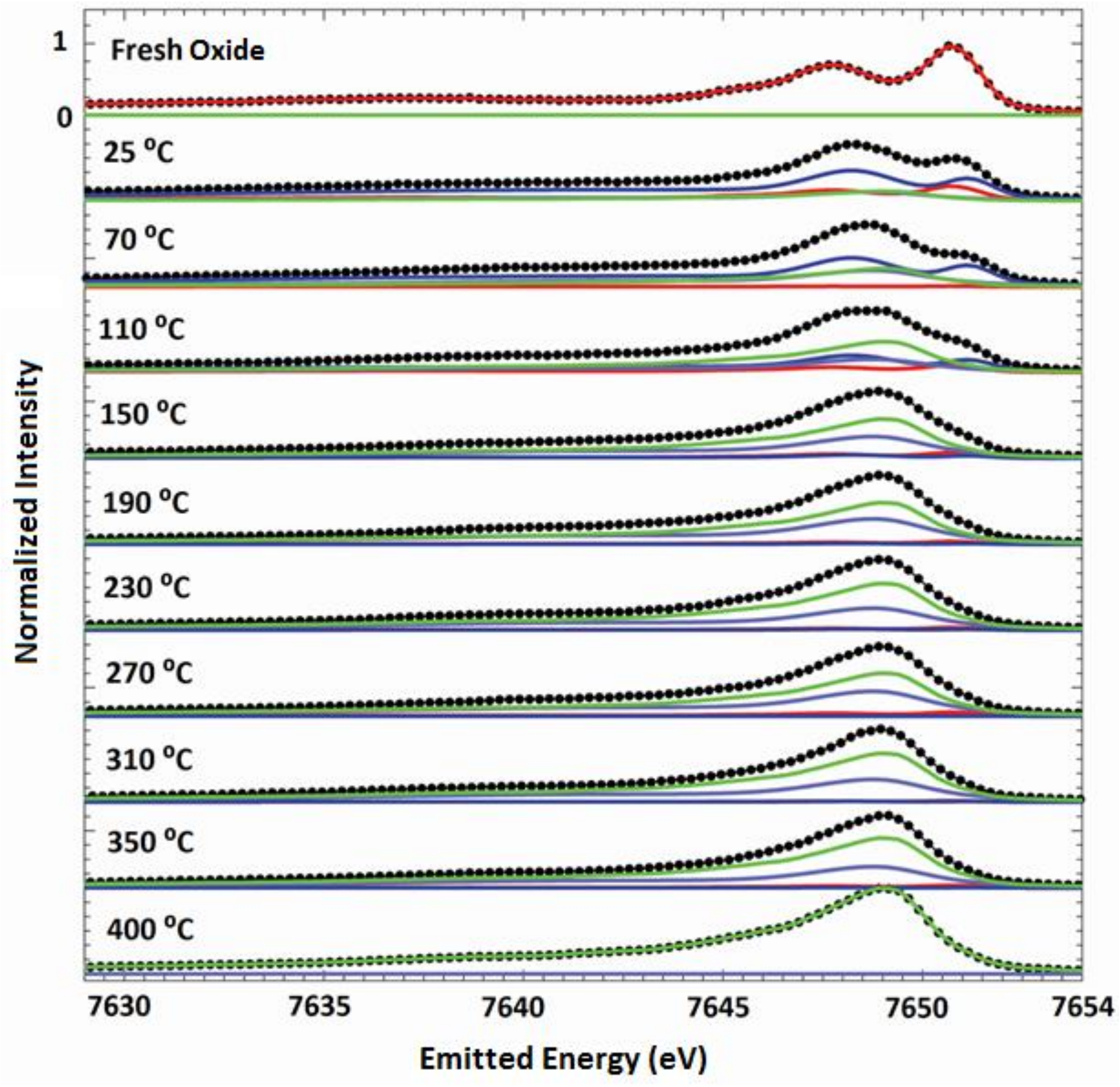

Figure S5 Series of Cobalt 1s3p RIXS spectra (black) obtained during the in-situ sulfurization of CoNiMo/Al_2O_3. The character of the freshly calcined sample is identified as 100% oxide (red) while the sulfided sample at 400°C shows a 100% sulfided character (green). De RIXS slices were obtained by exciting the sample at the pre-edge peak energy. In contrast to the CoMo/Al_2O_3 case, the fitting process revealed the presence of two intermediate species shown in blue and purple that remain present during the entire reaction. At 25°C approximately 80% of the oxide phase is converted to either the intermediate species. In Figure 8 the purple intermediate is plotted in yellow.

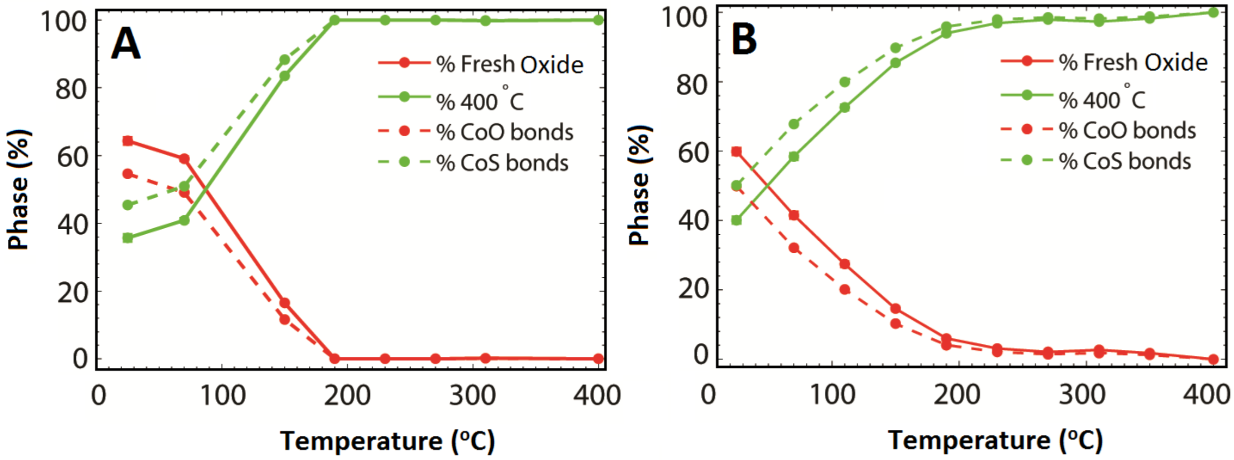

Figure S6 Composition expressed in terms of amount of the fresh sample spectrum and the data collected at 400^0C and in terms of Co-O and Co-S bonds as a function of temperature for CoMo/Al_2O_3 (A) and CoNiMo/Al_2O_3 (B).

Table S4 and table S5 list a summary of the chemical composition of various species during the in-situ sulfurization of CoMo/Al_2O_3 and CoNiMo/Al_2O_3 catalyst, respectively. These results were obtained by RIXS analysis of both samples.

Table S4 A summary of the chemical compositions for CoMo/Al_2O_3 as a function of temperature and under and atmosphere of 10% H_2S/H_2, expressed in terms of the relative amounts of the spectrum of the freshly calcined sample and the one collected at 400 °C. In addition, also the concentration and the respective standard deviation of the intermediate species is listed.

	**Oxide**		**Sulfide**		**Intermediate Species**	
	Avg	Std	Avg	Std	Avg	Std
**Freshly calcined**	1	0	0	0	0	0
**25**	0.6758	0.0539	0.0507	0.0743	0.2735	0.0785
**70**	0.609	0.0624	0.0564	0.0758	0.3346	0.0775
**150**	0.1298	0.0176	0.8685	0.0181	0.0017	0.0027
**190**	0.0454	0.0068	0.9544	0.0069	1.74E-04	8.28E-04
**230**	0.0257	0.0045	0.9449	0.0124	0.0295	0.0125
**270**	7.24E-04	0.0012	0.9993	0.0012	0	0
**310**	0.0324	0.0049	0.9676	0.0049	0	0
**400**	0	0	1	0	0	0

Table S5 A summary of the chemical compositions for $CoNiMo/Al_2O_3$ as a function of temperature and under and atmosphere of 10% H_2S/H_2, expressed in terms of the relative amounts of the spectrum of the freshly calcined sample and the data collected at 400 °C.

	**Fresh**		**Interm. 1(%)**		**Interm. 2(%)**		**400°C (%)**	
	Average	Std	Average	Std	Average	Std	Average	Std
**Freshly Calcined**	100	0	0	0	0	0	0	0
**25**	21.46	1.3	50.57	11.31	16.56	15.73	11.42	5.11
**70**	1.12	1.31	48.18	10.68	29.32	15	21.38	5.46
**110**	11.05	1.08	28.82	6.15	23.8	8.77	36.33	3.74
**150**	8.95	1.46	6.21	2.38	38.51	6.23	46.34	6.24
**190**	4.5	1.74	1.97	2.21	44.58	7.77	48.94	7.75
**230**	4.21	1.43	2.3	1.8	38.67	6.32	54.82	6.41
**270**	6.07	1.89	0.32	1.29	43.23	8.08	50.39	8.22
**310**	2.11	1.64	1.46	7.38	39.4	7.23	57.04	8.58
**350**	3.94	1.63	0.65	3.03	36.99	6.48	58.42	7.26
**400**	0	0	0	0	0	0	100	0
